# Deviation from the planned axis of three toric intraocular lenses

**DOI:** 10.1038/s41598-022-17811-x

**Published:** 2022-08-12

**Authors:** Shira Sheen-Ophir, Olga Reitblat, Adi Levy, Ehud I. Assia, Guy Kleinmann

**Affiliations:** 1 Ein-Tal Eye Center, 15 Habrzel St, 6971021 Tel Aviv, Israel; 2 Sackler Faculty of Medicine, Tel Aviv University, Tel Aviv, Israel; 3 Department of Ophthalmology, Hawke’s Bay Fallen Soldiers’ Memorial Hospital, Hastings, New Zealand; 4 Department of Ophthalmology, Rabin Medical Center, Petach Tikva, Israel; 5 Department of Ophthalmology, Meir Medical Center, Kfar Sava, Israel; 6 Department of Ophthalmology, E. Wolfson Medical Center, Holon, Israel

**Keywords:** Medical research, Optics and photonics

## Abstract

In this study, we retrospectively evaluated the deviation from the planned axis of 3 Toric intraocular lenses (TIOL). Included in the study 190 eyes, operated by two surgeons using two different manual marking techniques. The patients were implanted with either AcrySof IQ Toric SN6AT (Alcon) (n = 90), POD FT (PhysIOL) (n = 50), or TECNIS Symfony Toric (J&J) (n = 50). At least 1 month postoperatively, the IOL was photographed, and the axis was measured using a designed software. The difference between the planned and actual axis was defined as axis deviation. The effect of IOL type, astigmatism direction, and marking techniques on the average degree and direction of the IOL deviation were evaluated and compared. There was no significant difference in the average deviation between the IOLs (TECNIS Symfony: 4.03° ± 4.34, POD FT: 3.52° ± 3.38, and SN6AT: 4.24° ± 4.10), and its direction (55.8%, 39.0%, and 56.6% clockwise (CW) deviation, respectively). With the rule, astigmatism had significantly more CW deviation compared with against the rule and oblique astigmatism (64.3%, 43.8%, and 41.7%, respectively, P = 0.027), but the average deviation was similar. The marking techniques did not influence the degree or direction of the deviation.

## Introduction

Nowadays, cataract surgery has become a refractive surgery. With the development of toric intraocular lenses (TIOLs), surgeons can provide satisfactory refractive results as patient’s demand spectacle independence, even in cases of corneal astigmatism. Ferrer-Blasco et al. ^1^ found that 22.2% of the patients had corneal astigmatism of 1.5 D or more. Hoffer et al.^2^ reported mean corneal astigmatism of 1.0 D.

Optimal astigmatism correction with a TIOL requires accurate IOL alignment and rotational stability. The greater the IOL cylinder power, the greater the importance of correct alignment and stability of the lens. Deviation of a TIOL from its intended orientation decreases its corrective power, with approximately 3.3% loss of cylindrical power for every degree of misorientation^3^.

Our study aimed to compare the deviation from the planned axis of 3 types of TIOLs, AcrySof IQ Toric (Alcon Surgical Inc., Fort Worth, TX), POD FT (FineVision, PhysIOL inc., Liège, Belgium), TECNIS Symfony Toric, (Johnson & Johnson Vision, Santa Ana, CA), and to evaluate the effect of the eye characteristics, TIOL type, corneal astigmatism direction and surgeons marking techniques on the amount of deviation from the planned axis and its direction.

## Patients and methods

The medical charts of 190 eyes with a TIOL implantation during cataract surgery, operated by two surgeons using two different manual marking techniques, were retrospectively reviewed. The study adhered to the tenets of the Declaration of Helsinki, and all methods were carried out following the relevant guidelines and regulations. The Meir Medical Centre Ethics Committee approved all experimental protocols. The IRB of the Meir Medical Centre Ethics Committee waived the requirement for informed consent from the study subjects.

### Lenses

Three types of TIOLs were compared: SN6AT (Alcon Laboratories, Inc., Fort Worth, TX, USA), POD FT (PhysIOL, Liège, Belgium), and Symfony ZXT (Johnson & Johnson Vision, Santa Ana, CA, USA). The SN6AT AcrySof IQ Toric is monofocal toric, hydrophobic acrylic IOL. It has a 6.0 mm optic diameter, and its overall length is 13.0 mm. The FineVision POD FT is a trifocal toric hydrophilic IOL with an optic body diameter of 6.00 mm and an overall diameter of 11.40 mm. The ZXT Symfony Toric lens is an extended depth of focus, toric, foldable, hydrophobic acrylic 1-piece lens, with an optic diameter of 6 mm and an overall diameter of 13 mm.

### Lens selection

Preoperatively, all patients underwent measurements by the Lenstar LS 900, IOLMaster 500, Pentacam, and the Atlas topographer.

All biometric measurements were compatible with the strict validation criteria described by Warren Hill (Hill-RBF Calculator Version 2.0 https://rbfcalculator.com/online/index.html). The surgeon chose the implanted TIOL using the Barrett online toric calculator (https://ascrs.org/barrett-toric-calculator) and the IOL manufactures’ online calculators, (https://www.acrysoftoriccalculator.com/, https://www.physioltoric.eu/, https://www.tecnistoriccalc.com/). The TIOL power and placement axis were calculated using the Lenstar measurement when agreement between all devices was achieved. When no agreement was achieved between Lenstar and the IOL Master, the measurements that were closer to the axis of the topographer and tomographer were chosen for the IOL calculation. When no agreement was achieved between all devices toric IOL was not implanted. Additional adjustments were done according to the surgeon’s experience and preferences (e.g., surgical main incision site, posterior corneal astigmatism and astigmatism orientation).

### Surgical technique

Two experienced surgeons (EIA, GK) performed all the surgeries at Ein-Tal Eye Center, Tel-Aviv.

Ink marks were placed, preoperatively, at the limbus, while the patient's head was positioned behind the Haag-Streit (Köniz, Switzerland) slit lamps. The two surgeons used different manual marking techniques. One surgeon (EIA), marked the planned axis of the TIOL lens (Primary IOL marking) directly. The second surgeon (GK) marked 0, 180- and 270-degrees (Reference axis marking). The Incision and the IOL axis were marked as the first step of the surgery using the Mendez ring or other similar ring.

All surgical procedures performed through a small clear corneal incision (2.2–2.4 mm), using the phacoemulsification technique. After removing the cataract, the IOL was implanted into the capsular bag under an anterior chamber maintainer (ACM) and without an ophthalmic viscoelastic device (OVD). The IOL dialed into the planned axis and pushed gently back against the posterior capsule.

### Follow-up

**Figure 1:**
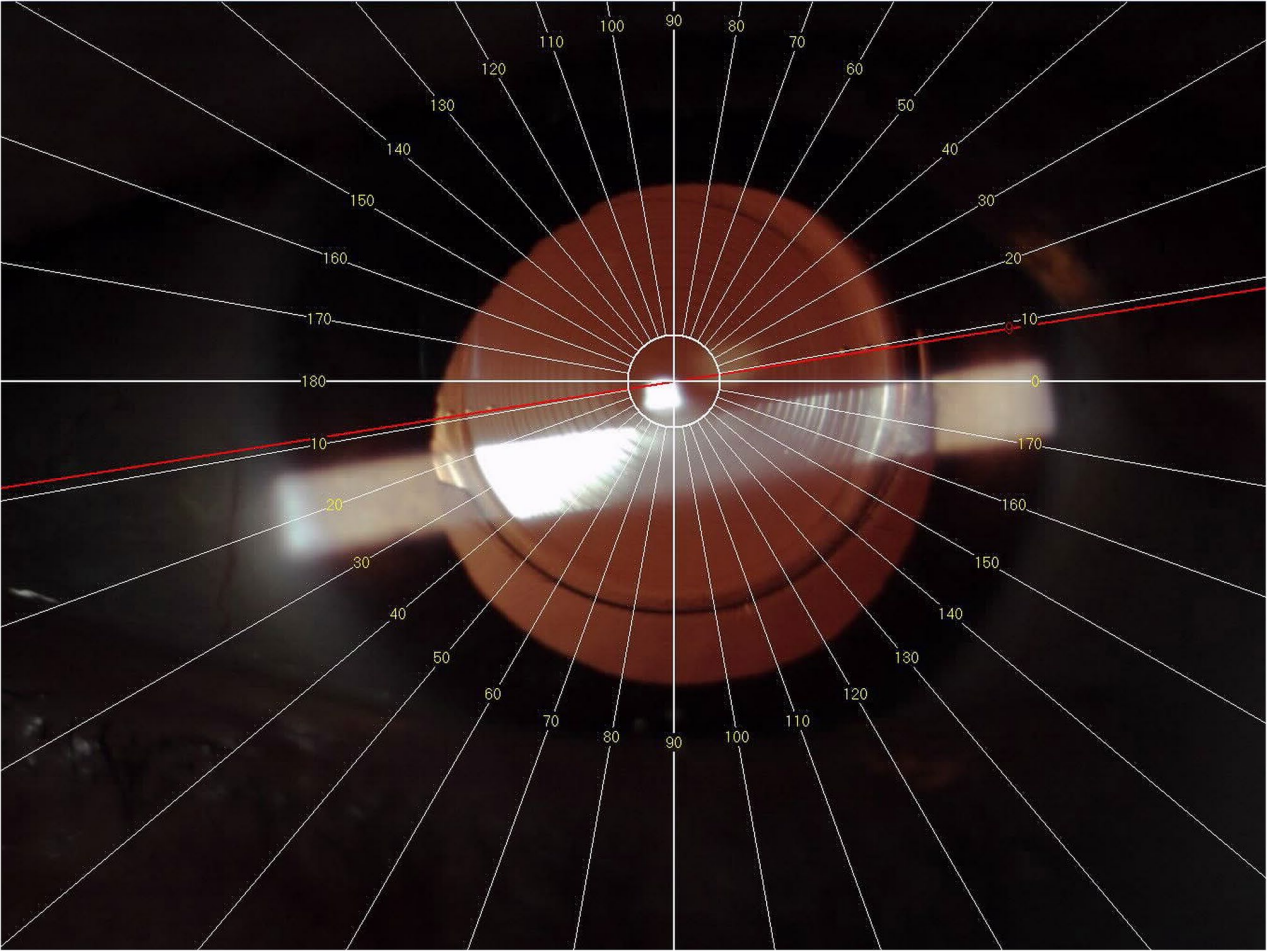
Example of a Retroillumination photo documentation of a TIOL position, using the Goniotrans software (Eventos Médicos y Sociales, SL). By dragging the radial line to the position of the TIOL axis mark on the angles pattern the position of the TIOL is determined.

Follow-up visits were 1 day, 1 week, and 1 month after the surgery. On the 1 month postoperative visits, a complete dilated examination and manifest refraction were performed. The TIOL position was detected, and retroillumination pictures were taken with a slit lamp digital color camera (CSO Elite Mega Digital Vision camera). The Goniotrans software (Eventos Médicos y Sociales, SL) was used to determine the TIOL axis. This software integrates virtual protractor over an eye picture, by dragging the radial line to the position of the toric lens axis marks on the angles pattern (Fig. 1)^7^.

### Data analysis

For the study outcome, we evaluated and compared the effect of the IOL type (cylinder and spherical power), surgeons marking techniques, and other eye characteristics such as corneal astigmatism (direction and power) axial length, and anterior chamber depth, on the IOL deviation and its direction. TIOL deviation was defined as the difference between the TIOL planned axis and the actual alignment axis measured at least 1 month after surgery.

### Statistical analysis

Statistical analyses were performed using SPSS (version 21.0, SPSS, Inc., Chicago, IL). Between-subjects design was applied, as specified in the editorial of the *Journal of Cataract and Refractive Surgery* on IOL studies^4^. Continuous variables were compared using a one-way analysis of variance (ANOVA) or the Kruskal–Wallis test, followed by post-hock tests, as indicated by a normality test (Shapiro–Wilk). Categorical variables were compared using the Pearson’s chi-square test. Pearson correlation was used to evaluate the correlation between axial length, anterior chamber depth, preoperative astigmatism, TIOL cylinder power and TIOL spherical power to the degree of rotation. A *P* value of less than 0.05 was considered statistically significant.

## Results

The study includes 190 eyes with toric IOL implantation: 90 eyes with SN6AT IOL, 50 with POD FT and 50 with ZXT. The patient’s’ demographics, preoperative biometric data, and the IOL characteristics are summarize in Table 1. No differences in the baseline characteristics and preoperative measurements were found between the three groups of TIOLs except for the corneal astigmatism, that was highest in the SN6AT group followed by ZXT and the POD FT groups (2.38D ± 0.78 vs. 1.69D ± 0.96 and 1.58D ± 0.85 respectively, P < 0.001), and the direction of the corneal astigmatism; in the SN6AT group there were more cases of with-the-rule (WTR) astigmatism (56.7%) while in the ZXT and POD FT TIOLs there were more cases of against-the-rule (ATR) astigmatism (46.0% and 56.0% respectively), P < 0.005. The toric IOL power was similarly distributed between the different types of TIOLs.

**Table 1:** Patient’s demographic, biometric data, and the implanted TIOLs.

	ZXT N = 50	POD FT N = 50	SN6AT N = 90	P value
Age (years), mean ± SD	68.1 ± 11.0	67.0 ± 9.0	66.3 ± 9.3	0.579
Ganger (% male)	56.4	39.4	49.3	0.354
Laterality (% RE)	56.0	60.0	54.4	0.816
Axial Length (mm), mean ± SD	24.32 ± 1.52	24.17 ± 1.41	24.70 ± 1.80	0.139
Average Keratometry (D), mean ± SD	43.63 ± 1.35	43.99 ± 1.42	44.23 ± 1.55	0.066
Absolute Astigmatism (D), mean ± SD	1.69 ± 0.96	1.58 ± 0.85	2.38 ± 0.78	< 0.001*
Centroid Astigmatism (D), mean ± SD	0.36 ± 1.18 @ 14	0.18 ± 1.12 @ 30	0.70 ± 1.50 @ 84	X = 0.788 Y = 0.007**
Astigmatism Direction (% WTR, ATR, OBL)	32.0, 46.0, 22.0	32.0, 56.0, 12.0	56.7, 35.6, 7.8	0.005
IOL (D), mean ± SD	19.25 ± 5.07	18.98 ± 4.21	17.84 ± 5.55	0.222
Toric Correction (D), mean ± SD	2.53 ± 1.35	2.46 ± 1.09	3.00 ± 1.23	0.018^¥^

Biometry measured by IOL-Master 500.

*RE* right eye, *D* diopters, *WTR* with-the-rule (60°–120°), *ATR* against-the-rule (0°–30°, 150°–180°), *OBL* oblique (30°–60°, 120°–150°).

*ZXT, POD FT < SN6AT.

**SN6AT < ZXT.

^¥^POD FT < SN6AT.

### IOL deviation

**Figure 2:**
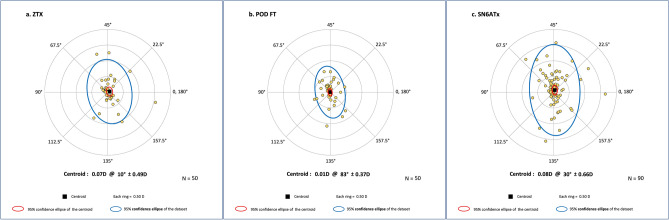
Double angle plots of the misalignment of: (**a**) ZXT (Johnson & Johnson Vision, Santa Ana, CA, USA), (**b**) POD FT (PhysIOL, Liège, Belgium) and (**c**) SN6ATx (Alcon Laboratories, Inc., Fort Worth, TX, USA) TIOLs. *D* dioptre, *TIOLs* toric intraocular lenses.

The average deviation from the planned axis was similar for the different IOLs (ZXT 4.030 ± 4.34, POD FT 3.520 ± 3.38, and SN6AT IOLs 4.240 ± 4.10, P = 0.588). No difference in the misalignment direction was found (55.8%, 39.0% and 56.6% clockwise [CW] deviation, respectively, P = 0.155), Table 2. Some of the lenses were precisely on planed axis (ZXT 14%, POD FT 18%, and SN6AT 7.8%). Double angle plots of the misalignment of each IOL model are presented in Fig. 2.

**Table 2:** The TIOL deviation and its direction of the different TIOLs.

	ZXT	POD FT	SN6AT	P value
misalignment (°), absolute mean ± SD [range]	4.03 ± 4.34 [0, 19]	3.52 ± 3.38 [0, 16]	4.24 ± 4.10 [0, 26]	0.588
CW misalignment, n (%*)	24 (55.8)	16 (39.0)	47 (56.6)	0.155
CCW misalignment, n (%*)	19 (44.2)	25 (61.0)	36 (43.4)	

*TIOL* toric intraocular lens, *CW* clockwise, *CCW* counterclockwise.

*IOLs that were precisely on the planned axis were excluded.

The corneal astigmatism direction (WTR, ATR, OBL) did not influence on the mean degree of misalignment (4.40 ± 4.38, 3.84 ± 3.84, and 3.13 ± 2.79, respectively) Table 3. WTR corneal astigmatism eyes had a statistically significant CW deviation percentage than ATR and oblique corneal astigmatism eyes (64.3%, 43.8%, and 41.7%, respectively, P = 0.027), Table 3.

**Table 3:** Influence of the astigmatism direction on the TIOL degree of deviation and its direction.

	WTR N = 83	ATR N = 83	OBL N = 24	P value
Absolute misalignment (°), mean ± SD [range]	4.40 ± 4.38 [0, 26]	3.84 ± 3.84 [0, 25]	3.13 ± 2.79 [1, 12]	0.345
CW misalignment, n (%*)	45 (64.3)	32 (43.8)	10 (41.7)	0.027
CCW misalignment, n (%*)	25 (35.7)	41 (56.2)	14 (58.3)	

*TIOL* toric intraocular lens, *CW* clockwise, *CCW* counterclockwise, *WTR* with-the-rule (60°–120°), *ATR* against-the-rule (0°–30°, 150°–180°), *OBL* oblique (30°–60°, 120°–150°).

*IOLs that were precisely on the planned axis were excluded.

The deviation of the TIOL from the planned axis divided to subgroup according to the degree of the deviation: < 1° (n = 23), 1°–5° (n = 102), 6°–10° (n = 53), > 10 (n = 12), Table 4. TIOL deviation of 1°–5° was the most common range of rotation for the three types of TIOLs. Deviation of 5° or less was more common in the oblique corneal astigmatism compared with ATR and WTR corneal astigmatism (83.3% versus 61.5% and 65.0%, respectively, P = 0.043). There was no significant difference between the operated eye (right or left), the IOL design, misalignment direction, and the marking techniques.

**Table 4:** The total deviation of the TIOL from the planned axis.

	< 1°	1°–5°	6°–10°	> 10°	P value
**Eye**					
RE	11.2%	56.1%	26.2%	6.5%	0.873
LE	13.3%	50.6%	30.1%	6.0%	
**TIOL model**					
SN6AT	7.8%	53.3%	34.4%	4.4%	0.304
POD FT	18.0%	54.0%	22.0%	6.0%	
ZXT	14.0%	54.0%	22.0%	10.0%	
**Corneal astigmatism direction**					
WTR	15.7%	45.8%	31.3%	7.2%	0.043
ATR	12.0%	53.0%	30.1%	4.8%	
OBL	0.0%	83.3%	8.3%	8.3%	
**Misalignment direction**					
CW	–	65.0%	30.0%	5.0%	0.460
CCW	–	57.5%	33.3%	9.2%	
**Surgeon**					
Reference axis marking	11.2%	56.2%	25.8%	6.7%	0.897
Primary IOL marking	12.9%	51.5%	29.7%	5.9%	

*RE* right eye, *LE* eye, *TIOL* toric intraocular lens, *CW* clockwise, *CCW* counterclockwise, *WTR* with-the-rule (60°–120°), *ATR* against-the-rule (0°–30°, 150°–180°), *OBL* oblique (30°–60°, 120°–150°).

No difference was found in the degree and direction of misalignment between the two surgeons marking techniques, Table 5.

**Table 5:** Influence of the marking technique on the TIOL deviation and its direction.

	Reference axis marking N = 89	Primary IOL marking N = 101	P value
Misalignment (°), mean ± SD [range]	3.80 ± 3.68 [0, 19]	4.17 ± 4.24 [0, 26]	0.530
CW misalignment, n (%*)	38 (48.1)	49 (55.7)	0.328
CCW misalignment*, n (%*)	41 (51.9)	39 (44.3)	

No correlation was found between TIOL deviation and axial length (r = 0.062, P = 0.397), anterior chamber depth (r = 0.055, P = 0.453), preoperative astigmatism (r = 0.026, P = 0.719), TIOL cylinder power (r = − 0.054, P = 0.455) and TIOL spherical power (r = − 0.028, P = 0.700).

*TIOL* toric intraocular lens, *CW* clockwise, *CCW* counterclockwise.

*IOLs that were precisely on the planned axis were excluded.

### Visual outcomes

The refractive visual outcomes evaluated at least 1 month after the surgery are present in Table 6. The mean spherical equivalent (SEQ) in the SN6AT group was more myopic than in the ZXT and POD FT groups due to some eyes targeted for monovision, with one eye aimed for near vision resulting in expected myopic outcomes. SN6AT TIOLs were found to have the highest residual mean absolute refractive cylinder. A significant statistical difference was found compared to POD FT (P < 0.001). Mean postoperative centroid residual astigmatism was higher in the ZXT group, with a WTR orientation, compared with the SN6AT and ZXT groups (P = 0.005, for X-axis).

**Table 6:** Postoperative manifest refraction outcomes.

	ZXT	POD FT	SN6AT	P value
SEQ (D), mean ± SD	− 0.37 ± 0.24	− 0.16 ± 0.26	− 0.69 ± 0.66 *	< 0.001**
Absolute Astigmatism (D), mean ± SD	0.42 ± 0.28	0.31 ± 0.22	0.52 ± 0.38	0.001^¥^
Centroid Astigmatism (D), mean ± SD @ axis	0.17 ± 0.33 @ 100	0.06 ± 0.26 @ 35	0.06 ± 0.44 @ 180	X = 0.005^§^ Y = 0.301

*D* diopters, *SEQ* spherical equivalent.

*Including monovision.

**SN6AT < ZXT, POD FT.

^¥^POD FT < SN6AT.

^§^ZXT < SN6AT.

## Discussion

Toric IOLs designed to correct corneal astigmatism during cataract surgery and increase spectacle independence. It is even more critical with multifocal IOLs, which require minimal residual astigmatism for their total effectivity. Successful astigmatism correction requires accurate measurements and calculation of the power, and axis of corneal astigmatism, precise marking and alignment of the TIOL during the surgery, and excellent rationale stability after the TIOL implantation. Every degree of deviation from the desired axis reduces the effectivity of the TIOL by 3.3%^3^. In this study, we found similar and low deviations from the planned axis of 3 common TIOL: SN6AT (Alcon Laboratories, Inc., Fort Worth, TX, USA), POD FT (PhysIOL, Liège, Belgium), and Symfony ZXT (Johnson & Johnson Vision, Santa Ana, CA, USA).

Inoue et al.^5^ found that most of the postoperative TIOL rotation occurs within the first hour. Similarly, Lee and associates^6^ suggested that postoperative rotation occurs very soon after the surgery since the alignment results for patients examined at least 1 h postoperatively or the following day were identical. Our study evaluated the TIOL position at least 1 month postoperatively using slit-lamp photography and purpose-designed software, assuming no further deviation should be expected. It is important to note that we measured the total deviation from the planned axis, including marking and alignments inaccuracies and postoperative IOL rotation. Our findings were similar to the reports of most studies reporting 5 degrees or less of misalignment ^7–13^. We found that the three investigated IOLs had an excellent and similar small rates of deviation from the planned axis (ZXT = 4.03° ± 4.34°, POD FT = 3.52° ± 3.38°, SN6AT = 4.24° ± 4.10°). There was also no difference in the deviation CW or CCW direction. The Eye characteristic, axial length, anterior chamber depth, the direction of corneal astigmatism, and the method of the manual marking technique (reference plane or planned IOL position) were found to not influence on the degree of the deviation. The AcrySof SN6AT and the Symfony ZXT are 13.0 mm diameter lenses designed with C-loop haptics, In contrast the FineVision POD FT is an 11.40 mm diameter lens designed with double C-loop 4-point-fixation. One should expect that due to the smaller diameter, a higher rate of rotation is expected^14^. The stability provided by the unique double C-loop haptics POD FT TIOLs design may compensate for the smaller diameter and explain our finding^15^.

Lee et al.^6^ compared the rotational stability of the Acrysof and Tecnis monofocal TIOL (aside from the diffractive optic, the monofocal, and the extended depth of focus Tecnis TIOLs retain the same single piece, hydrophobic acrylic design). Contrary to our results, they found that the Tecnis TIOL had significantly higher overall rotation, predominantly CCW. As opposed to Lee et al. findings, we found a similar deviation and no predominantly for the deviation direction. CW deviation was found in 56.6% of the eyes implanted with the SN6AT and 55.8% of eyes implanted with the ZXT.

Schartmüller et al. studied the rotational stability of Vivinex XY1 IOL, a single-piece hydrophobic intraocular lens, in 122 eyes. They found significantly higher CW TIOL with no correlation to the corneal astigmatism axis^16^. Ruhswurm et al.^17^ measured IOL rotation and found significantly greater rotation with the vertical axis.

We found that CW deviation was significantly more common in WTR corneal astigmatism without correlation to the degree of TIOL deviation. The underlying factors for the CW rotation are unclear.

Till et al.^18^ observed that the oblique axis was the most unstable. In our study, eyes with oblique corneal astigmatism showed better results with more eyes within 5 degrees or less of the planned axis compared with eyes with WRT and ATR corneal astigmatism.

Several studies have demonstrated the accuracy of manual marking. The three-step marking method is pretty accurate, with a total error of 4.9° ± 2.1° in toric IOL alignment^19^. Popp et al. evaluated four different marking techniques: coaxial slit beam, bubble marker, pendular marker, and tonometer marker. All methods except the tonometer marker gave accurate results with rotational misalignment between 1.8° and 2.9°^20^. We also did not find a significant difference in the TIOL deviation degree and direction when using two different manual marking techniques. Several studies that investigated predictors for TIOL rotation found, contrary to our findings, that longer axial length and lower spherical IOL power are positive predictors for TIOL rotation. These may be due to the larger size of the capsular bag expected in these cases enabling the TIOL to rotate more easily^6,13,21–24^.

The weaknesses of this study include its retrospective design and lack of randomization. Secondly, PIOL axis measurement technics available today are not free of error. Viestenz et al. demonstrated that slight deviations might be the result of cyclorotation of the eye during standardized photography^25^. However, the measurement method used in this study is based on software analysis designed for this purpose. It is highly reproducible and similar to methods used in other studies^26,27^. It is important to notice that we measured the total deviation from the planned axis, including marking and alignments inaccuracies and postoperative IOL rotation. This provides information on real-life expectations and results of TIOLs.

In conclusion, our results suggest that the three investigated TIOL models yield excellent and similar deviations from the planned axis. The IOL design and material had no influence on the magnitude and direction of the toric IOL deviation. We found significantly more CW deviation in WTR corneal astigmatism than in ATR and oblique corneal astigmatism, with no difference in its magnitude. No difference was found when comparing the two surgeons’ manual marking techniques in the degree and direction of the deviation.

## Data Availability

The datasets used and or analysed during the current study are available from the corresponding author upon reasonable request. Inclusion criteria were: (1) preoperative biometric measurements by IOLMaster-500 (Carl Zeiss Meditec AG, Jena, Germany) and Lenstar LS 900 (Haag-Streit, Koeniz, Switzerland), (2) corneal topography by the Atlas topographer (Carl Zeiss Meditec) and Pentacam scheimpflug camera (Oculus, Optikgerate GmbH, Wetzlar, Germany), (3) implantation of SN6AT, POD FT or ZXT TIOLs, IOLs (4) uneventful cataract surgery and (4) at least 1 month follow-up exam including postoperative photographic documentation of the TIOL axis. Exclusion criteria were perioperative complications and incomplete data or follow-up.
